# Use of a calcium tracer to detect stone increments in a rat calcium oxalate xenoplantation model

**DOI:** 10.3892/etm.2013.1233

**Published:** 2013-07-24

**Authors:** SHUO WANG, QINGQUAN XU, XIAOBO HUANG, JINGXING LIN, JINXING WANG, XIAOFENG WANG

**Affiliations:** 1 Department of Urology, Peking University People’s Hospital, Beijing 100044;; 2 Institute of Geology, Chinese Academy of Geological Sciences, Beijing 100037, P.R. China

**Keywords:** fluorochromes, calcium oxalation, rat, xenoplantation

## Abstract

The majority of urinary stones have been observed to grow by circular increments in the clinic and in animal studies. However, the mechanism of stone formation has not yet been elucidated. Marking the stone at specific time-points during the growth of the stone is likely to enable the clarification of the mechanisms behind lithogenesis. The objective of this study was to evaluate the role and efficacy of calcium-tracing fluorescence in the labeling of stone lamination in a rat calcium oxalate xenoplantation model. In the rat calcium oxalate xenoplantation model, human renal stone particles, extracted by percutaneous nephrolithotomy, were xenoplanted into the bladders of Wistar rats in a sterile manner. The rats received 1% ethylene glycol in their drinking water, starting from the day following the stone xenoplantation. Two weeks subsequent to this, three calcium-tracing fluorochromes, alizarin complexone, calcein and xylenol orange were administered by intraperitoneal injection. The newly-formed bladder stones were cut into slices and examined using light and fluorescence microscopy. The newly-formed bladder stones had a large variance in size, and circular increments were observed in the sections of the stones. The stones were successfully labeled with calcein and alizarin complexone, although calcein labeling provided superior results. However, the use of xylenol orange did not result in clear labeling. The calcium-tracing fluorochromes, calcein and alizarin complexone may be effectively used to label stone lamination in rat models.

## Introduction

Urolithiasis is a frequently occurring global disease. The formation of stones in the urinary tract affects 5–10% of the population in Europe and the United States (1,2), and the annual incidence of stone formation in the industrialized world is generally considered to be 1,500–2,000 cases per million (3). Furthermore, the prevalence of nephrolithiasis appears to have increased in the last quarter of the twentieth century (4). The prevalence of stone disease shows a geographical variability, with China being an area with a high prevalence (5). At present, renal stones are the most common form of urolithiasis in China (6). Although there is currently little epidemiological information available regarding urinary stones in China, clinical data has shown an increasing trend in the incidence of urinary stones (6).

At present, extracorporal shock wave lithotripsy (ESWL) and percutaneous nephrolithotomy (PCNL) are the primary treatments for renal stones. Circular stone increments have been frequently observed during PCNL procedures in our clinical practice. Similar observations have been apparent in the ear stones of fish, known as otoliths. Otolith micro-structure investigative techniques may be applicable to the examination of the age and growth of the juvenile chum salmon *Oncorhynchus keta*, which inhabits coastal waters (7).

There are, at present, few techniques that may be used to study stone growth, particularly in the evaluation of drug efficacy in medical expulsive therapy. Approximately 80% of kidney stones contain calcium, and the majority of calcium stones consist primarily of calcium oxalate (8,9). In the present study, stone circular increments were labeled using calcium-tracing fluorochromes in a rat calcium oxalate xeno-plantation model.

## Materials and methods

### Establishment of the rat calcium oxalate xenoplantation model

Stone particles were extracted by PCNL from one male patient with renal stones. Informed consent from the patient was obtained prior to the study. One stone particle was sent for analysis by infrared spectroscopy. The result showed that it was predominantly composed of calcium oxalate. Following this, other particles were selected, cut with a blunt instrument into sections with a diameter of 2–3 mm, weighed and maintained in a sterile environment, prior to use.

Eight-week-old male Wistar rats (Vital River Laboratory Animal Technology Co., Ltd., Beijing, China), weighing 250–300 g, were housed in a specific pathogen-free (SPF) environment. All animals had free access to drinking water and regular chow every day, and were kept under a controlled 12-h light/dark cycle at 22±2°C. All animal experimentation was performed in accordance with the Chinese Home Office-approved guidelines, and was approved by the Animal Care Committee of Peking University People’s Hospital (Beijing, China).

The rats were anesthetized by intraperitoneal injection of sodium pentobarbital [50 mg/kg body weight (bw)] and the bladder was exposed by a suprapubic incision. Following this, a 4–5 mm incision was made at the top of bladder and one prepared human stone particle was inserted. The bladder and suprapubic incision were closed respectively. Ethylene glycol (EG) was supplied in the drinking water at a final concentration of 1% from the second day (day 1) postoperatively for 4 weeks.

### Fluorochrome application

The method of fluorochrome administration was modified from previous studies (10). Briefly, three fluorescent chromophores, calcein, alizarin complexone and xylenol orange, were administered by intraperitoneal injection from day 15, postoperatively. The dosages used were 15, 30 and 90 mg/kg bw, respectively (10). Two protocols were used in the fluorochrome application. Protocol 1 entailed each fluorescent chromophore being intraperitoneally injected twice a week for 2 weeks (continuous labeling), while protocol 2 entailed the sequential injection of calcein, alizarin complexone and xylenol on each consecutive day for 1 week (sequential labeling). All fluorochromes were purchased from the Beijing Chemical Reagent Company (Beijing, China) and were sterilized by filtration, prior to use.

Four weeks after EG was supplied in the drinking water, the rats were sacrificed with an intraperitoneal injection of an overdose of sodium pentobarbital. The kidneys and urinary bladder were dissected and the kidneys were dehydrated in a graded ethanol series and embedded in paraffin.

The bladder stones were harvested, weighed and maintained in 75% ethanol for 24 h, prior to the stones being embedded in autopolymerizing resin and sectioned transversely with a diamond wire saw in order to select the best section plane. Sectioned blocks were then fixed to a glass slide with a thermoplastic glue and polished successively using a 1,200 grit sandpaper and a mix of alumina polishing compounds (3, 1 and 0.3 *μ*m) with a small volume of water, until it was possible to observe the core clearly under a transmitted light microscope. Thermoplastic glue, which softens when heated, enabled the block to be turned over so that was possible to polish the other side and for the core to be approached cautiously.

### Detection of renal stone formation

Renal stone formation was assessed using von Kossa histochemical staining. Briefly, 5-*μ*m-thick cross sections of the paraffin-embedded rat kidneys were deparaffinized and placed in distilled water, prior to being exposed to 2% silver nitrate for 60 min. Subsequent to being washed in 5% sodium thiosulfate for 5 min, the sections were counterstained with neutral red and examined under a light microscope. The 5-*μ*m cross sections were also observed under a fluorescence microscope.

### Bladder stone analysis

Bladder stone analysis was undertaken using a confocal laser scanning microscope (Leica TCS-SP2; Leica Microsystems, Wetzlar, Germany) fitted with spectrophotometers for emission band wavelength selection. The calcein and alizarin complexone were excited with the 476 and 530 nm laser lines from an argon laser, with the laser intensity set at 9% of the available power. For the visualization of calcein and alizarin complexone, the emission windows were set at 496–505 and 530–580 nm, respectively. Any areas of interest that were well-focused and exhibited sufficient reflection or fluorescence were captured using the Leica Confocal software.

## Results

### Renal stone confirmation

**Figure 1.**
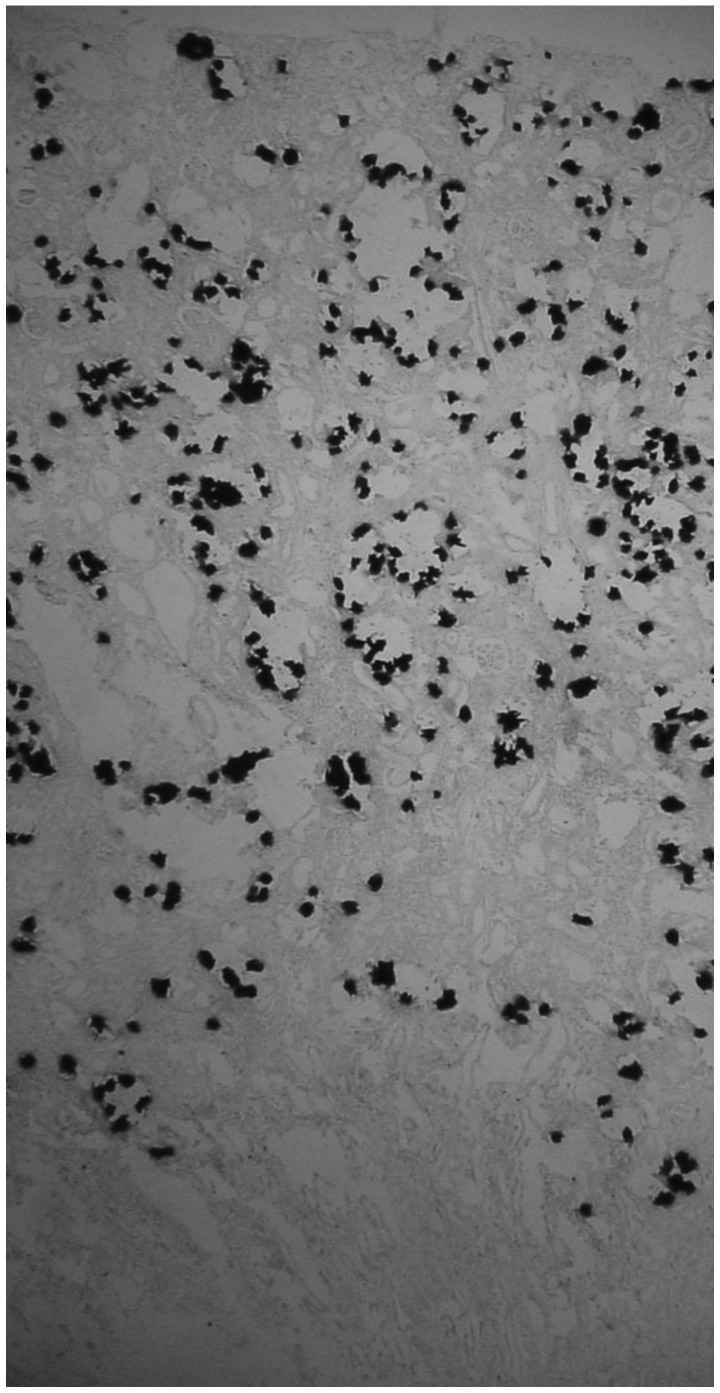
Results of renal von Kossa histochemical staining. The stones were predominantly formed in the renal cortex. Magnification, ×40.

Kidney stone formation was confirmed by von Kossa histochemical staining in all seven rats. The stones were predominantly formed in the renal tubules located at the border between the renal cortex and the medulla (Fig. 1).

### Bladder stone regrowth

**Figure 2.**
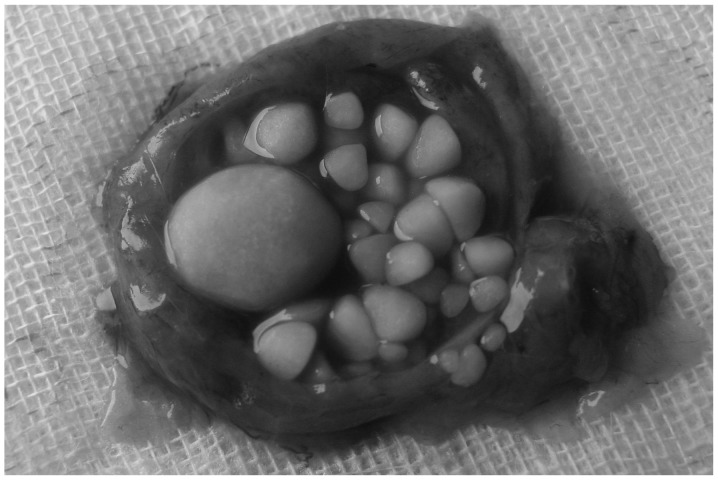
Stones grown in a rat bladder by xenoplanting a human renal stone particle. There was a large variation in the size of the stones.

The bladder stones showed a large variation in the size following regrowth in the rats (Fig. 2). The average stone weight was 0.14 g (range, 0.01–0.61 g).

### Stone microscopic analysis

**Figure 3.**
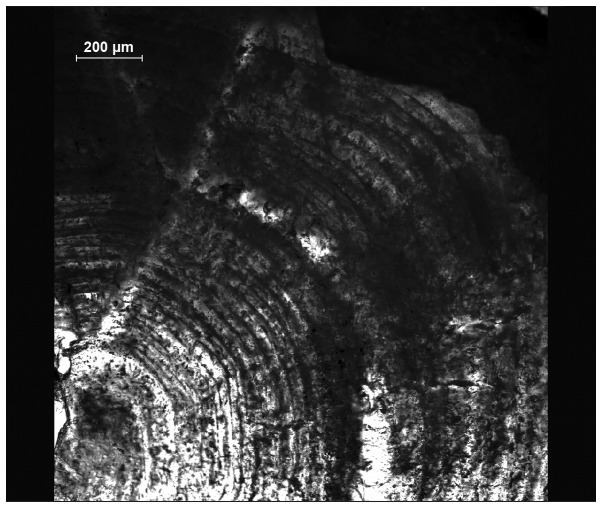
Circular stone increments of a bladder stone. Observed under a transmitted light microscope, the bladder stones were revealed to form in a circular manner.

**Figure 4.**
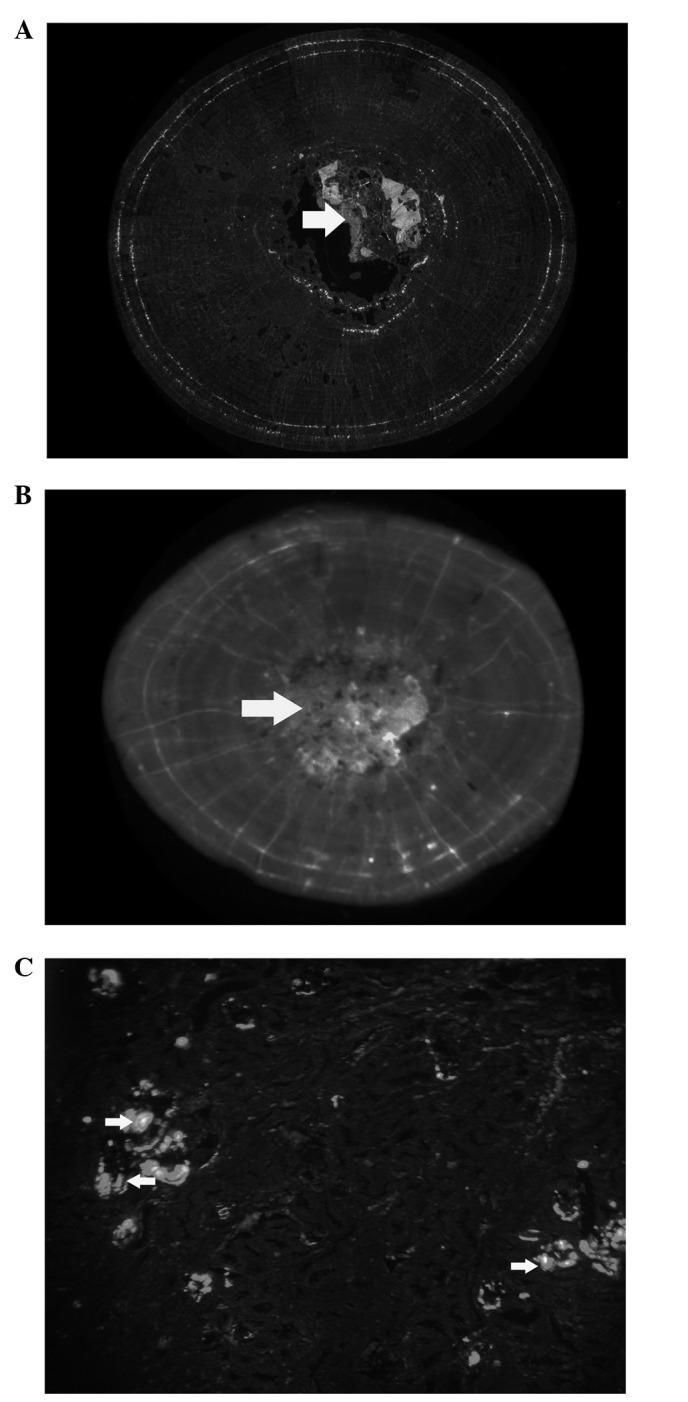
Calcium tracer labeling results under a fluorescence microscope. (A) Calcein labeling showed clear white bands in the bladder stone. The arrow shows the human stone core xenoplanted into the rat bladder. (B) Alizarine complexone labeling showed a white band in the bladder stone. The arrow shows the human stone core xenoplanted into the rat bladder. (C) Dark and light marks (arrows) were also observed in the rat kidney using sequential labeling. Magnification, ×40.

The circular bladder stone increments were observed under the laser scanning confocal microscope (Fig. 3). Under fluorescence emission, clear colored marks were apparent in the sections that had been prepared by continuous labeling with calcein and alizarin complexone (Fig. 4A and B); however xylenol orange did not produce the same effect. Green and red labeling was also observed in the renal sections prepared by sequential labeling (Fig. 4C).

## Discussion

Nephrolithiasis is a particularly common clinical condition in the industrialized world. Up to 15% of Caucasian males and 6% of all females are likely to have at least one stone during their lifetime, and half of these individuals will experience recurrent episodes (11,12). During recent decades, the incidence of the disease has appeared to increase, although the exact cause of the disease has yet to be elucidated. Among the various types of renal stones, those composed of calcium oxalate are by far the most prevalent, accounting for 75% of all stones (13).

With regard to calcium oxalate crystallization, there are numerous complexities. Crystal nucleation, growth, aggregation and retention are considered to be the fundamental steps in stone formation. The final step of the process is poorly understood; however, the assembly of crystalline material is a necessary factor in stone formation. Circular increments have been observed in the majority of renal stones during PCNL procedures. Similar stone increments were apparent in the rat calcium oxalate xenoplantation model in the present study. This phenomenon has been described in detail in previous studies (14,15).

To define the time-course of stone growth in the clinic is difficult, due to the challenge presented in marking the stone at a specific time-point. An identical problem is encountered in studies with animal models. Enabling the effective labeling of the stone at specific time-points is likely to be beneficial for clinical and animal model studies. Calcium-tracing fluorochromes are widely used in investigations concerning the process of mineralization, including otoliths, tooth growth and bone remodeling (10,16–18). The fluorochromes that are frequently used are alizarin complexone, calcein, xylenol orange and oxytetracycline. The sites labeled by these fluorochromes are revealed as different colors under the fluorescence microscope. In the present study, clear circular labeling was observed with intraperitoneal injections of alizarin complexone and calcein, although not with xylenol orange. By labeling the stone at a specific time, alizarin complexone and calcein may be used to evaluate stone growth. In a previous study, Figueiredo *et al* applied the calcium-binding fluorescence probes, Osteosense 680 and Osteosense 750, in the identification of calculi in the urinary tract. In the study, the Osteosense 680 and Osteosense 750 probes demonstrated a variety of binding affinities for different types of stones. It was concluded that the improved visualization of these stones was likely to reduce the difficulties encountered in endoscopic procedures, decrease the risk of complications and increase the chance of rendering the patient stone-free (19).

Animal models are frequently used in the study of urolithiasis, and, as such, there are numerous animal models of urolithiasis (20). The rat calcium oxalate model, induced by ethylene glycol, is frequently used. Ethylene glycol administration has been demonstrated to result in an increase in urinary oxalate levels and calcium oxalate supersaturation, and to induce calcium oxalate crystalluria with a decreased calcium level (14,20). Since human urolithogenesis is different to that in the rat calcium oxalate model, it is not certain whether calcium tracers are likely to lead to the desired results in human urolithiasis labeling. Furthermore, the size of the bladder stones demonstrated a large variance in the present study, which makes this xenoplantation model unsuitable for urinary stone growth studies.

In conclusion, calcein and alizarin complexone effectively labeled the stone increments in the present rat calcium oxalate xenoplantation model. This technique may potentially be used to evaluate the efficacy of medical expulsive therapy by marking the stone increments at specific time-points and monitoring the rate of stone growth between two specific time-points.
